# Endophytic Bacteria Isolated from Citrus Plants for Biological Control of Citrus Canker in Lime Plants

**DOI:** 10.21315/tlsr2019.30.1.5

**Published:** 2019-01-31

**Authors:** Orawan Daungfu, Somchit Youpensuk, Saisamorn Lumyong

**Affiliations:** 1 Department of Biology, Faculty of Science, Chiang Mai University, Chiang Mai 50200, Thailand; 2 Center of Excellence in Bioresources for Agriculture, Industry and Medicine, Faculty of Science, Chiang Mai University, Chiang Mai 50200, Thailand

**Keywords:** Biocontrol, Citrus Canker, Endophytic Bacteria, Lime

## Abstract

Citrus canker caused by *Xanthomonas citri* subsp. *citri* is a disease affecting the yield and fruit quality of lime (*Citrus aurantiifolia*). This research investigated endophytic bacteria obtained from six healthy *Citrus* spp. to inhibit the pathogen and to control citrus canker on lime plants. Numbers of the endophytic bacteria isolated from *C. aurantifolia*, *C. hystrix*, *C. maxima*, *C. nobilis*, *C. reticulata* and *C. sinensis* were 28, 25, 29, 42, 12 and 34 isolates, respectively. The selected endophytic bacteria that were effective against *X. citri* subsp. *citri* were *Bacillus amyloliquefaciens* LE109, *B. subtilis* LE24 and *B. tequilensis* PO80. The optimum culture medium for an antagonistic effect on the pathogen in *B. amyloliquefaciens* LE109 and *B. tequilensis* PO80 was yeast extract peptone dextrose broth, and in *B. subtilis* LE24 was modified soluble starch broth. To control citrus canker in lime, young expanded leaves of lime plants were aseptically punctured and inoculated with 30 μl of bacterial suspension of the pathogen (10^8^ CFU/ml in 0.85% NaCl) per punctured location. After the pathogenic inoculation for 24 h, the leaves were then inoculated with 30 μl of the selected endophytic bacteria (10^8^ CFU/ml in 0.85% NaCl), and treated with 30 μl of the culture media containing bioactive compounds produced by the selected endophytic bacteria. The leaves inoculated with cell suspensions of *B. amyloliquefaciens* LE109 or *B. subtilis* LE24 could completely control citrus canker. However, the leaves inoculated with *B. tequilensis* PO80 displayed 10% disease incidence. Additionally, the leaves treated with the crude bioactive compounds of *B. amyloliquefaciens* LE109 or *B. subtilis* LE24 could completely control citrus canker. Notably, the leaves treated with the crude bioactive compounds of *B. tequilensis* PO80 displayed 5% disease incidence. The results of this study showed that the *Bacillus* strains play important roles in the biocontrol of citrus canker in lime.

## INTRODUCTION

Lime (*Citrus aurantiifolia* Swingle) is an important fruit tree that is commercially grown in Thailand. The primary problem associated with growing lime trees is the occurrence of citrus canker (Asiatic citrus canker), which is caused by *Xanthomonas citri* subsp. *citri* (synonyms: *X. axonopodis* pv. *citri*, *X. campestris* pv. *citri*, *X. citri* pv. *citri*) (Schaad *et al*. 2006; Jalan *et al*. 2013). The pathogen causes symptoms on leaves, fruits and twigs of lime plants. Symptoms of citrus canker include round spots that become brown and corky and are sunken in the centre with water-soaked margins surrounded by yellow chlorotic halos. The disease on lime plants causes defoliation, twig dieback and premature fruit drop (Cernadas & Benedetti 2009; Zhang & Meng 2011). The disease results in economic losses in terms of low quality and productivity of lime fruits and the costs for the disease control. Some growers applied chemical pesticides such as spraying copper compounds for control citrus canker. The use of chemical pesticides to control citrus canker can cause negative impacts on humans and the environment. Many growers have switched their farming methods to organic systems.

Biological control of plant diseases by antagonistic microorganisms has been considered using in organic farming systems. Additionally, consumers express increasing concerns about health and have demanded a higher degree of quality in plant products. Some endophytic microorganisms can be used to control pathogens and promote the growth of the host plants (Sturz *et al*. 2000; Gaiero *et al*. 2013). Endophytic microorganisms exist inside plant tissues without causing disease symptoms for the host plants (Schulz & Boyle 2006). Microorganisms can enter into plant tissues via the stomata, lenticels, wounds, roots and germinating radicles. Some bacteria are generally found in the soil and are also associated with plants as endophytic bacteria, which are known to be both Gram-negative and Gram-positive (Mahaffee & Kloepper 1997; Senthilkumar *et al*. 2011). Many bacteria were reported as effective biocontrol agents of plant pathogens (Bacon & Hinton 2002; Mahadtanapuk et al. 2007; Ren *et al*. 2013; Soares *et al*. 2016) Objective of this research was to investigate endophytic bacteria obtained from various healthy *Citrus* spp. in the control of citrus canker in lime plants.

## MATERIALS AND METHODS

### Isolation and Detection of Bacteria Causing Lime Canker

Six samples of leaf and fruit of lime plants displaying citrus canker symptoms were washed under running tap water. The lesions on each sample were cut into approximately 5 × 5 mm pieces with a sterilized scalpel and immersed in 0.6% NaOCl for 3 min followed by three rinses in sterile distilled water. The surface-sterilized samples and 2 ml of sterile 0.85% NaCl were combined and crushed in a sterilized mortar. The bacterial suspension was streaked in a sterilized loop on nutrient glucose agar (NGA) and then incubated at room temperature (28°C–32°C) for 2 to 3 days. Morphological characteristics regarding the shape and colour of colonies, Gram staining, cell shape and cell arrangement were determined. The bacteria were streaked on NGA plates to confirm purity. The presence of bacteria was determined according to some biochemical tests described in Holt *et al*. (1994) and Schaad *et al*. (2001). Molecular identification of the bacteria was also performed according to the method noted below.

### Pathogenicity Test of Pathogenic Bacteria

The bacteria isolated from citrus canker were determined for pathogenicity towards citrus canker on the detached leaves of lime plants. Young expanded leaves of lime plants were washed under running tap water, surface-sterilized in 0.6% NaOCl for 3 min and rinsed three times in sterile distilled water. The surface-sterilized leaves were then aseptically punctured, creating five wounds at each location (two locations on each leaf) and placed in a moist chamber. The wounds of the leaves were inoculated with 30 μl of bacterial suspension (10^8^ CFU/ml of 0.85% NaCl). For the negative control, 30 μl of 0.85% NaCl without bacterial cells were deposited onto the wounds. The leaves were incubated at room temperature under light (12 h/day) to observe disease progression.

### Isolation of Endophytic Bacteria Obtained from Citrus Plants

Endophytic bacteria were isolated from healthy *Citrus* spp. of lime (*C. aurantiifolia* Swingle), kaffir lime (*C. hystrix* DC.), pomelo (*C. maxima* Merr.), mandarin orange (*C. reticulata* Blanco), sweet orange (*C. sinensis* Pers.) and tangerine orange (*C. nobilis* Lour.) in the Chiang Mai Province of northern Thailand. Twenty-four samples were obtained from leaves, young twigs and roots from each of the citrus plants. The samples were cut into pieces (5 × 5 mm for leaves and 5 mm in length for young twigs and roots) and immersed in 0.6% NaOCl for 3 min and 70% ethanol for 1 min. The specimens were then washed three times in sterile distilled water. The surface-sterilized samples were placed on sterile tissue paper to absorb any water. The samples were then placed on nutrient agar (NA) in Petri dishes and incubated at room temperature for 48 h. Bacterial colonies on NA were streaked on new NA plates. Single colonies of the bacterial isolates were stored on NA slants at 4°C and in nutrient broth (NB) mixed with 25% glycerol at −20°C for further study.

### Screening and Measuring of Endophytic Bacteria for the Inhibition of *X. citri* subsp. *citri*

Screening for the ability of endophytic bacteria to inhibit *X. citri* subsp. *citri* was performed using a dual culture technique. Cell suspension of *X. citri* subsp. *citri* was swabbed on the surface of NA, and then each endophytic bacteria specimen was streaked with four lines (2 cm long) that were then coupled with opposites at a 1 cm distance from the four edges of the plates and incubated at room temperature for 48 h to check inhibition zones around the four streaks of each endophytic bacteria specimen on the NA in each Petri dish.

The endophytic bacteria that displayed antagonistic effects on *X. citri* subsp. *citri* were confirmed for *X. citri* subsp. *citri* inhibition using the agar well diffusion method. After swabbing *X. citri* subsp. *citri* on the NA plates, four wells (Ø 5 mm) were made on each plate of the NA, and then 30 μl (10^8^ CFU/ml) of each endophytic bacteria specimen that was cultured in NB for 48 h was added into the four wells of each of the NA plates. To create a negative control for the NA plates, NB was added to the wells without endophytic bacteria. All of the NA plates were incubated at room temperature for 48 h to measure the diameter of the inhibition zones and thus select effective endophytic bacteria.

### Molecular Identification of Selected Effective Bacteria

Three isolates of the endophytic bacteria (LE24, PO80 and LE109 shown in Table 3) were selected as effective bacteria to inhibit *X. citri* subsp. *citri*. The selected bacteria were determined for colony morphologies, Gram staining, bacterial shapes, cell arrangements and endospore formation. DNA extraction of the selected bacteria was performed by homogenisation. Colonies of the selected bacteria were crushed in a 1.5 ml Eppendorf tube, and 100 μl of buffer A (100 mM Tris-HCl, 1M KCl and 10 mM EDTA) was added. The bacteria were then vortex mixed for 1 min, incubated at 94°C for 15 min and vortexed again for 1 min. The mixtures in the Eppendorf tubes were centrifuged at 12,000 rpm for 10 min. The supernatants were transferred to new Eppendorf tubes to be used as DNA templates. The DNA specimens were amplified from 16S rRNA genes using a KOD FX kit. Universal primers of 27f, 5′-AGAGTTTGATCCTGGCTCAG-3′ and 1492r, 5′-GGTTACCTTGTTACGACTT-3′ were used for PCR products. DNA sequences of the PCR products were compared with the sequences in GenBank by the BLAST (Basic Local Alignment Search Tool) programme (NCBI, http://blast.ncbi.nlm.nih.gov/). Alignment of the DNA sequences was carried out using MUltiple Sequence Comparison by Log-Expectation (MUSCLE) program. Phylogenetic analyses were performed using the maximum likelihood methods with 1000 bootstrap replications from the MEGA 6 programme.

### Investigation of Optimum Culture Media of Selected Endophytic Bacteria to Assess the Efficiency of *X. citri* subsp. *citri* inhibition

The selected effective endophytic bacteria were cultured in four culture media, including NB, nutrient glucose broth (NGB), modified soluble starch broth (MSSB) and yeast extract peptone dextrose broth (YEPDB). The inhibition of *X. citri* subsp. *citri* was performed using the agar well diffusion method. After swabbing *X. citri* subsp. *citri* on the NA plates, four wells (Ø 5 mm) were made on each plate of the NA and then 30 μl (10^8^ CFU/ml) of each endophytic bacteria specimen that had been cultured in each culture media for 72 h was added to the four wells of each of the NA plates. For the negative control of the NA plates, the culture was added to the wells with culture media without culturing the endophytic bacteria. All of the culture plates were incubated at room temperature for 48 h to measure the diameter of the inhibition zones.

### Extraction and Evaluation of Crude Bioactive Compounds from Selected Endophytic Bacteria

The selected bacteria were cultured in 5 L of their optimum culture media in an orbital shaker for 96 h. The bacterial cells were separated from the culture media by centrifugation at 10,000 rpm for 10 min. The culture media were extracted by mixing with ethyl acetate 1:1 (v/v). The supernatants of the extracts were collected by centrifugation at 10,000 rpm for 10 min. The extraction process was repeated three times. The extracts were dried in a rotary evaporator at 45°C, and the dried extracts of the secondary metabolites were weighed. The dried extracts were dissolved in 1 ml of methanol and stored at 4°C for further use. Concentrations of the crude extracts were evaluated for the minimum inhibitory concentration (MIC) against the growth of *X. citri* subsp. *citri* using the agar well diffusion method.

The crude extracts of the bioactive compounds were separated by thin-layer chromatography on TLC silica gel Gf_245_ plates with solvents of CH_3_Cl: MeOH: H_2_O (65: 25: 4, v/v/v). The separated components on the TLC plates were evaluated under ultraviolet light (254 nm), placed in a chamber containing iodine crystals and sprayed with ninhydrin reagent to detect the colour bands of the secondary metabolite components.

### Investigation of Effective Endophytic Bacteria on the Inhibition of Citrus Canker in Lime Plants

The bacterial cells and the culture media containing bioactive compounds produced by the selected bacterial isolates LE24, PO80 and LE109 were investigated regarding their inhibition of citrus canker on grafted lime plants in a greenhouse. The bacterial cells were separated from the optimum culture media by centrifugation at 8,000 rpm for 10 min. Each treatment was performed with five lime plants on four leaves per plant. Young expanded leaves of the lime plants were surface-sterilized by being wiped with 70% ethanol, after which the leaves were aseptically punctured by creating five wounds at each puncture location (two locations on each leaf). The wounds were inoculated with 30 μl of bacterial suspension of *X. citri* subsp. *citri* (10^8^ CFU/ml in 0.85% NaCl). For the non-inoculated control, 30 μl of 0.85% NaCl without the bacterial cells were deposited onto the wounds. After inoculation of the pathogen for 24 h, 30 μl of the bacterial suspensions of each LE24, PO80 and LE109 (10^8^ CFU/ml in 0.85% NaCl), as well as 30 μl of the culture media containing bioactive compounds produced by the selected bacteria were dropped onto the wounds of each plant. Disease incidence of citrus canker was observed for one month after inoculation. Percentage of the disease incidence of each treatment was evaluated from number of the diseased leaves of the treatment multiplied by 100 and divided by number of the total assessed leaves of the treatment.

## RESULTS AND DISCUSSION

### Characterisation and Pathogenicity Test of Bacteria Isolated from Citrus Canker on Lime

Colonies of the bacteria isolated from citrus canker on the leaves and fruits of lime plants were circular, convex, muciod and had smooth margins. The colour of the colonies was creamy yellow on the NGA, which was in accordance with the general characteristics of *X. citri* subsp. *citri*. The yellow colour is due to the xanthomonadin produced by *X. citri* subsp. *citri* (Schaad *et al*. 2001). Characteristics of the bacteria isolated from citrus canker on lime were presented in Table 1. According to the general characteristics and biochemical tests, the bacteria isolated from citrus canker on lime was identified as putative *X. citri* subsp. *citri*. [Schaad *et al*. 2001; European and Mediterranean Plant Protection Organization (EPPO) 2005; International Standards for Phytosanitary Measures (ISPM) 2014]. However, the range of cell size of the isolated strain (*X. citri* subsp. *citri* CM-TH) was little longer than the cell size of *X. citri* subsp. *citri* mentioned by ISPM (2014).

**Figure 1:**
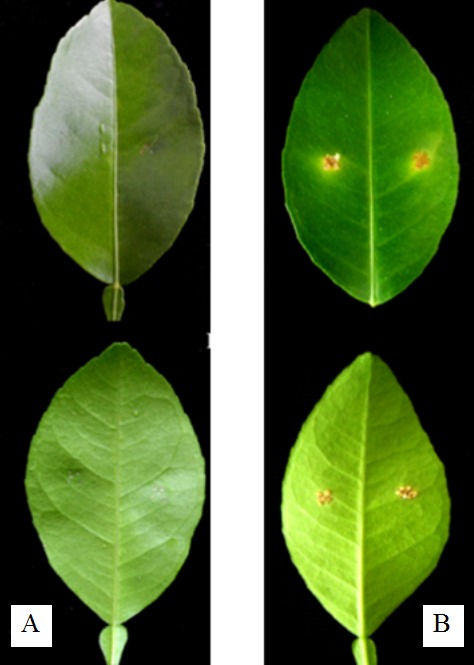
*Leaves of Citrus aurantiifolia* (upper side and lower side) after two weeks of incubation in a moist chamber (A) without bacterial inoculation and (B) with inoculation using the bacteria isolated from lime with canker disease.

**Figure 2:**
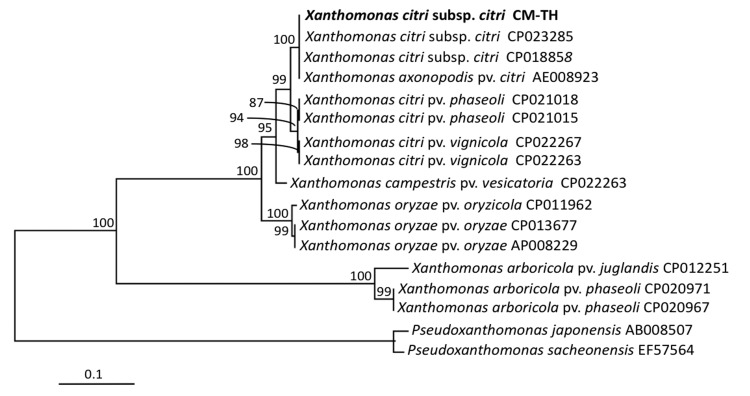
Phylogenetic analysis of the isolated pathogen from citrus canker in lime (CM-TH) was obtained using maximum likelihood method. Scale bar represent substitutions per nucleotide position.

For pathogenicity test, the wounds on the detached lime leaves began to show symptoms at one week after inoculation of the bacteria isolated from citrus canker. The symptoms of citrus canker included a brown and corky appearance that was surrounded by a yellow chlorotic halo after two weeks. However, in the negative control, the leaves with wounds that were dropped with 30 μl of 0.85% NaCl without the bacterial cells did not show any symptoms of citrus canker (Fig. 1). EPPO (2005) reported that the lesions of citrus canker developed 7–14 days after inoculation of the pathogen on intact or detached leaves. *X. citri* subsp. *citri* is the most widespread agent of Asiatic citrus canker on economic citrus plants (Schaad *et al*. 2006). The bacteria isolated from citrus canker in lime was confirmed as *X. citri* subsp. *citri* using molecular identification (Fig. 2). The GenBank accession number of 16S rDNA of *X. citri* subsp. *citri* CM-TH (the isolated pathogen of citrus canker of this study) was MG980566.

### Endophytic Bacteria Isolated from Citrus Plants and Screening of the Bacteria for the Inhibition of *X. citri* subsp. *citri*

One hundred seventy (170) isolates of endophytic bacteria were collected from the six healthy *Citrus* spp. Most of the isolates were Gram-positive rods of 132 isolates (77.65%), and the rest were Gram-negative rods of 38 isolates (22.35%). Numbers of the endophytic bacteria isolated from *C. aurantifolia*, *C. hystrix*, *C. maxima*, *C. nobilis*, *C. reticulata* and *C. sinensis* were 28, 25, 29, 42, 12 and 34 isolates, respectively (Table 2). The isolated bacteria were about 45%, 30% and 25% of the total isolates that were collected from young twigs, leaves and roots of the six *Citrus* spp., respectively.

**Figure 3:**
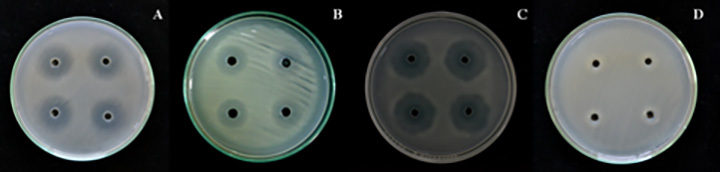
Inhibition zones of the selected endophytic bacteria against *X. citri* subsp. *citri* using the agar well diffusion method on nutrient agar for (A) *Bacillus* LE 24, (B) *Bacillus* PO 80, (C) *Bacillus* LE 109 and (D) Control treatment.

Only 10 isolates or about 6% of 170 isolates of the endophytic bacteria could inhibit *X. citri* subsp. *citri* in a dual culture technique. All of the 10 isolates were Gram-positive, rod-shaped and endospore-forming bacteria in which the cells were single or arranged in chains. Their colonies were a whitish cream on NA, and they belong to the genus *Bacillus* (Table 3). Some strains of *Bacillus* spp. have been reported to have antagonistic effects on some plant pathogens or to induce systemic resistance of the host plants (Krause *et al*. 2003; Szczech & Shoda 2006; Choudhary & Johri 2009). After screening the endophytic bacteria against *X. citri* subsp. *citri*, the 10 isolates were confirmed for antagonistic effects on *X. citri* subsp. *citri* by the agar well diffusion method. The three effective isolates, which had the highest zones of inhibition by the agar well diffusion method against *X. citri* subsp. *citri*, were *Bacillus* LE24, *Bacillus* PO80 and *Bacillus* LE109 (Fig. 3). Both *Bacillus* LE24 and *Bacillus* LE109 were isolated from healthy lime plants (*C. aurantifolia*), and *Bacillus* PO80 was isolated from healthy pomelo plants (*C. maxima*). The endophytic bacteria were selected to inhibit the growth of *X. citri* subsp. *citri* and control citrus canker disease of the lime plants.

### Identification of the Effective Endophytic Bacteria

**Figure 4:**
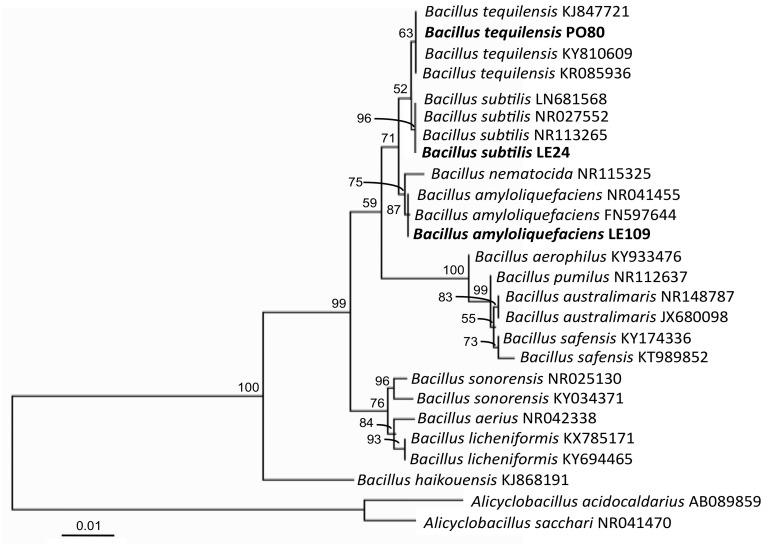
Phylogenetic analysis of the three isolates of *Bacillus subtilis* LE24, *B. tequilensis* PO80 and *B. amyloliquefaciens* LE109 was obtained using the maximum likelihood method. Scale bar represent substitutions per nucleotide position.

The isolate *Bacillus* LE24 had nearly round colonies on NA. The cell size of *Bacillus* LE24 was approximately 3.0–5.0 × 0.4–1.0 μm. The endospore position was sub-terminal. *Bacillus* LE24 produced acid from glucose, sucrose and mannitol. However, *Bacillus* LE24 did not produce acid from lactose and galactose. The specimens were catalase-positive and oxidase-positive, while the indole production of *Bacillus* LE24 was negative. These biochemical properties were in accordance with the properties of *B. subtilis* (Holt *et al*. 1994). Molecular identification of the 16S rDNA sequence of *Bacillus* LE24 was most similar to the group of *B. subtilis* (Fig. 4).

Colonies of *Bacillus* PO80 on NA were nearly round to lemon-shaped. The cell size of *Bacillus* PO80 was approximately 4.0–5.0 × 0.8–0.9 μm and catalase-positive. The endospore position was central. The indole production and oxidase test of *Bacillus* PO80 were positive. *Bacillus* PO80 produced acid from glucose, galactose, sucrose, lactose and mannitol. Molecular identification was similar to *B. tequilensis* (Fig. 4). The biochemical properties of *Bacillus* PO80 in this study were also similar to those of *B. tequilensis* sp. nov., which were described by Gatson *et al*. (2006).

The molecular identification of the 16S rDNA sequence of *Bacillus* LE109 belonged to the group *B. amyloliquefaciens* (Fig. 4). The colony morphology of *Bacillus* LE109 was irregularly shaped on NA. The cell size of *Bacillus* LE109 was approximately 3.0–5.0 × 0.6–0.7 μm, and this sample was catalase-positive. The endospore position was sub-terminal. *Bacillus* LE109 produced acid from glucose, sucrose, lactose and mannitol but did not produce acid from galactose. *Bacillus* LE109 was oxidase-positive, and the indole production was negative, which was in accordance with the properties of *B. amyloliquefaciens* (Priest *et al*. 1987). The GenBank accession number of 16S rDNA of *B*. *subtilis* LE24, *B*. *amyloliquefaciens* LE109 and *B*. *tequilensis* PO80 obtained in this study were MG980567, MG980568 and MG980569, respectively.

### Optimum Culture Media of the Selected Endophytic Bacteria for the Efficient Inhibition of *X. citri* subsp. *citri*

The optimum culture media of *B. subtilis* LE24, *B. tequilensis* PO80 and *B. amyloliquefaciens* LE109 for the inhibition of *X. citri* subsp. *citri* were MSSB, YEPDB and YEPDB, respectively. Their optimum culture media gave significantly higher clear zones than when cultured in the other media (Table 4). The medium of MSSB contains yeast extract, soluble starch, glucose, CaCl_3_ and trace element solution. While, the medium of YEPDB contains yeast extract, peptone and glucose. The components in the culture media may be suitable to enable the *Bacillus* species to produce some bioactive compounds, which could inhibit the growth of *X. citri* subsp. *citri*. Both the culture media of MSSB and YEPDB were composed of yeast extract, which contains B vitamins, amino acids, peptides and carbohydrates. Todar (2012) reported that B vitamins played important roles as coenzymes in many metabolic processes to fulfill biosynthesis requirements in the bacterial cells.

### Evaluation of Crude Extracts of Bioactive Compounds from Selected Endophytic Bacteria

The dry weights of the crude extracts collected from 5 L of *B. subtilis* LE24, *B. tequilensis* PO80 and *B. amyloliquefaciens* LE109 cultured in MSSB, YEPDB and YEPDB were 420, 380 and 460 mg, respectively. The crude extracts were evaluated for MIC against the growth of *X. citri* subsp. *citri* using the agar well diffusion method. The MIC of the crude extracts of the bioactive compounds obtained from *B. subtilis* LE24, *B. tequilensis* PO80 and *B. amyloliquefaciens* LE109 were 0.3, 1.5 and 0.3 mg/ml, respectively. The antagonistic compounds obtained from the bacteria may be antibiotics or toxins that could inhibit the growth of *X. citri* subsp. *citri*. The crude extracts of the bioactive compounds produced by *B. subtilis* LE24 and *B. amyloliquefaciens* LE109 were effective in very low concentration compared with the crude extract of the bioactive compounds produced by *B. tequilensis* PO80.

In the TLC investigation, the separated components on the TLC plates included the evaluated colour bands under ultraviolet light (254 nm), reaction with iodine vapor and reaction with ninhydrin reagent. The colour bands of the three evaluation methods showed similar patterns of the R_f_ (retention factor) values. TLC plates under ultraviolet light of *B. subtilis* LE24 revealed four dark spots at the R_f_ values of 0.63, 0.70, 0.80 and 0.89. However, the TLC plate of *B. tequilensis* PO80 showed two spots at the R_f_ values of 0.70 and 0.80, while the TLC plate of *B. amyloliquefaciens* LE109 had three spots at the R_f_ values of 0.70, 0.74 and 0.89. There were at least four, two and three components in the crude extract of *B. subtilis* LE24, *B. tequilensis* PO80 and *B. amyloliquefaciens* LE109, respectively. Reaction with iodine vapour of all of the separated components on the TLC plates produced brown spots, which revealed the presence of lipids and organic compounds in the components. All of the components on the TLC plates also revealed purple spots when sprayed with ninhydrin reagent. The reactions revealed that the components also contained amino acids (Touchstone 1992). The bioactive compounds were within the group of lipopeptides. The fractions on the TLC plates of *B. subtilis* LE24, *B. tequilensis* PO80 and *B. amyloliquefaciens* LE109, which appeared at the same R_f_ values, may be the same as those of the bioactive compounds. Some strains of *B. subtilis* and *B. amyloliquefaciens* produced bioactive compounds of cyclic lipopeptides, such as surfactins, fengycins and iturins, which could inhibit some bacterial and fungal pathogens (Szczech & Shoda, 2006; Mahadtanapuk *et al*. 2007; Torres *et al*. 2016). Chen *et al*. (2009) reported that *B. amyloliquefaciens* FZB42 produced difficidin and bacilysin, which were efficient in controlling fire blight disease caused by *Erwinia amylovora*. In this experiment, the crude extracts of the bioactive compounds may be categorised into the group of cyclic lipopeptides.

### Disease Incidence of Citrus Canker on the Lime Plants

The three isolates of *B. subtilis* LE24, *B. tequilensis* PO80 and *B. amyloliquefaciens* LE109 were studied for their inhibition of citrus canker disease in lime plants. After inoculation of *X. citri* subsp. *citri* on the leaves of grafted lime plants in a greenhouse, all of the leaves inoculated with only the pathogen cells showed symptoms of citrus canker (100% disease incidence) within three weeks. However, the leaves inoculated with *X. citri* subsp. *citri* for 24 h and then inoculated with cell suspensions (10^8^ CFU/ml) of *B. subtilis* LE24 or *B. amyloliquefaciens* LE109 did not display symptoms of citrus canker. Moreover, the leaves treated with crude bioactive compounds of *B. subtilis* LE24 cultured in MSSB and the leaves treated with crude bioactive compounds of *B. amyloliquefaciens* LE109 cultured in YEPDB did not display symptoms of citrus canker. However, the treatments inoculated with cells of *B. tequilensis* PO80 displayed citrus canker with 10% disease incidence, and the leaves treated with crude bioactive compounds of *B. tequilensis* PO80 cultured in YEPDB displayed citrus canker with 5% disease incidence (Table 5). Therefore, *B. subtilis* LE24 and *B. amyloliquefaciens* LE109 were the most effective strains in controlling citrus canker disease in lime. The disease control by inoculation of the bacterial cells may result from both competition with the pathogen for growth and the bacteria may produce bioactive compounds that inhibit the growth of *X. citri* subsp. *citri* on the lime plants. The effective bacteria, which were endophytic bacteria isolated from citrus plants, have the ability to multiply inside the host plant tissues. The *Bacillus* spp. can form endospores that can survive in the environment when applied to control plant diseases. Kalita *et al*. (1996) reported that *Bacillus subtilis* isolated from the phylloplane of lemon cv. Assam lemon could inhibit growth of *Xanthomonas campestris* pv. *citri* and could reduce citrus canker incidence under field conditions. Soares *et al*. (2016) reported that *B. Amyloliquefaciens* strain C6c isolated from English ivy (*Hedera helix* L.) could enhance growth and control disease of the host plant.

## CONCLUSION

In this study, *B. subtilis* LE24, *B. amyloliquefaciens* LE109 and *B. tequilensis* PO80, which are endophytic bacteria isolated from healthy citrus plants, displayed an ability to inhibit the growth of *X. citri* subsp. *citri*. The most effective strains used to control citrus canker disease on lime were *B. subtilis* LE24 and *B. amyloliquefaciens* LE109. The benefits of the endophytic bacteria include the ability to multiply inside the host plant tissues. Moreover, the genus *Bacillus* forms endospores that can survive in the environment when applied to control plant diseases. The *Bacillus* strains thus play important roles in the biological control of citrus canker disease on lime plants.

## Figures and Tables

**Table 1:** Characteristics of the pathogenic bacteria isolated from citrus canker.

Characteristics	The bacteria isolated from citrus canker on lime	*Xanthomonas citri* subsp. citri*
Gram staining	Negative	Negative
Cell shape	Rod	Rod
Cell size (μm)	2.0–2.5 × 0.5–0.75	1.5–2.0 × 0.5–0.75
Catalase	+	+
Oxidase	−	− or weak
Nitrate reduction	−	−
Urease	−	−
Gelatin liquefaction	+	+
Starch hydrolysis	+	+
Casein hydrolysis	+	+

* (Schaad et al. 2001; EPPO 2005; ISPM 2014)

**Table 2:** Endophytic bacteria isolated from various parts of citrus plants.

Citrus plants	Isolate numbers of endophytic bacteria			

	Young twigs*	Leaves	Roots	Total isolates
*C. aurantifolia*	12	7	9	28
*C. hystrix*	9	11	5	25
*C. maxima*	11	8	10	29
*C. nobilis*	24	10	8	42
*C. reticulata*	4	5	3	12
*C. sinensis*	17	10	7	34

Total	77	51	42	170

* Parts of growing branches of the citrus plants, which are still green.

**Table 3:** Inhibition zones of the endophytic bacteria isolated from citrus plants against *X. citri* subsp. *citri* by agar well diffusion method on nutrient agar.

*Citrus* species	Bacterial isolates	Diameter of inhibition zones (mm)
*C. aurantifolia*	*Bacillus* LE24	11.8 ± 0.5^b^
*C. maxima*	*Bacillus* PO28	4.3 ± 0.2^g^
*C. aurantifolia*	*Bacillus* LE32	7.8 ± 0.4^e^
*C. aurantifolia*	*Bacillus* LE59	6.1 ± 0.2^f^
*C. hystrix*	*Bacillus* KL66	6.5 ± 0.2^f^
*C. sinensis*	*Bacillus* SO70	4.1 ± 0.5^g^
*C. aurantifolia*	*Bacillus* LE76	8.3 ± 0.2^d^
*C. maxima*	*Bacillus* PO80	10.6 ± 0.5^b^
*C. aurantifolia*	*Bacillus* LE105	9.0 ± 0.4^c^
*C. aurantifolia*	*Bacillus* LE109	14.3 ± 0.3^a^

*Note:* The isolates with letter LE, PO, KL and SO were the endophytic bacteria isolated from lime (*C. aurantifolia*), pomelo (*C. maxima*), kaffir lime (*C. hystrix*) and sweet orange (*C. sinensis*) respectively. Diameter of inhibition zones with + standard error of means (n = 4), and different superscript letters indicated significant differences (*P* ≤ 0.05) according to Duncan’ multiple range test.

**Table 4:** Different culture media of the selected endophytic bacteria for efficiency on inhibition of *X. citri* subsp. *citri*.

Culture media	Diameter of inhibition zones (mm)		

	*B. subtilis* LE24	*B. tequilensis* PO80	*B. amyloliquefaciens* LE109
NB	10.3^c^	15.4^b^	15.8^b^
NGB	18.5^b^	16.1^b^	17.3^b^
YEPDB	12.1^c^	22.8^a^	25.3^a^
MSSB	24.4^a^	11.2^c^	7.0^d^

*Note:* Means of diameter of inhibition zones with different superscript letters indicated significant differences (*P* ≤ 0.05) according to Duncan’ multiple range test, NB: nutrient broth, NGB: nutrient glucose broth, YEPDB: yeast extract peptone dextrose broth, MSSB: modified soluble starch broth

**Table 5:** Disease incidence of citrus canker on the lime plants in a greenhouse after inoculation for one month.

Treatment	No. of diseased leaves	No. of total assessed leaves	Disease incidence (%)
Cell suspension*			
*B. subtilis* LE24	0	20	0
*B. tequilensis* PO80	2	20	10
*B. amyloliquefaciens* LE109	0	20	0
Bioactive compounds of the bacteria**			
*B. subtilis* LE24	0	20	0
*B. tequilensis* PO80	1	20	5
*B. amyloliquefaciens* LE109	0	20	0

Control treatment	20	20	100

*Note:* The leaves inoculated with *X. citri* subsp. *citri* (3×10^6^ CFU/punctured location) for 24 h and then inoculated with cell suspensions of the bacteria or bioactive compounds containing in the culture media.

* 30 μl of the bacterial suspension (108 CFU/ml in 0.85% NaCl),

** 30 μl of bioactive compounds containing in the culture media produced by the bacteria
